# Uncovering the genetic architecture of ME/CFS: a precision approach reveals impact of rare monogenic variation

**DOI:** 10.1186/s12967-025-07586-w

**Published:** 2025-12-24

**Authors:** Camille L. Birch, Brandon M. Wilk, Manavalan Gajapathy, Shaurita D. Hutchins, Gurpreet Kaur, Donna M. Brown, Tarun K. K. Mamidi, Kathleen S. Hodgin, Alp Turgut, Jarred W. Younger, Elizabeth A. Worthey

**Affiliations:** 1 Center for Computational Genomics and Data Science, Department of Genetics, 701 20th St S, Birmingham, AL 35233 USA; 2 Department of Psychology, University of Alabama-Birmingham, 1300 University Blvd Suite 233, Birmingham, AL 35294 USA

**Keywords:** Myalgic Encephalomyelitis/Chronic fatigue syndrome, Precision medicine, Transcriptomics, Genome sequencing, Convergence, Phenotype modeling, Molecular diagnostics

## Abstract

**Background:**

Myalgic encephalomyelitis/chronic fatigue syndrome (ME/CFS) is a disabling and heterogeneous disorder lacking validated biomarkers or targeted therapies. Clinical variability and elusive pathophysiology hinder progress toward effective diagnostics and treatment. Core symptoms include persistent fatigue, post-exertional malaise, unrefreshing sleep, cognitive dysfunction, and pain. We tested whether an individualized, “n-of-1” genomic and transcriptomic framework combined with comprehensive, participant-informed phenotyping could reveal molecular signatures unique to each patient.

**Methods:**

Clinical-grade whole-genome sequencing was conducted in 31 affected individuals from 25 families, with RNA-seq performed on a subset (16 affected, 7 unaffected) using blood samples. Machine-learning assisted variant triage, transcript-aware damage prediction, and expert review identified pathogenic or likely pathogenic variants in 8 of 25 probands (32%) and 12 of 31 affected individuals (39%).

**Results:**

Findings revealed marked genetic heterogeneity, including large-effect rare and more common variants. Implicated pathways included ATP generation, oxidative phosphorylation, fatty acid oxidation; regulation of glycolysis, amino acid and lipid turnover; ion and solute homeostasis; synaptic signaling, excitability, oxygen transport, and muscle integrity, resilience, and post-exertional recovery; previously implicated processes. Plausible modifiers influencing disease onset, severity, and relapsing–remitting patterns and possibly explaining intrafamilial variability and inconsistent findings across studies, were also identified. Despite gene-level diversity, downstream effects converged on impaired energy production, reduced stress resilience, and vulnerability to post-exertional metabolic failure; disruptions consistent with core ME/CFS symptoms of exertional intolerance, cognitive fog, and fatigue.

**Conclusions:**

Our findings support the hypothesis that at least a subset of ME/CFS cases represent distinct molecular disorders that converge on shared physiological pathways. Validation in larger, more diverse cohorts will be essential to test this hypothesis and establish generalizability, but increase size alone is unlikely to resolve causation in a disorder defined by rarity, heterogeneity, and molecular complexity. We suggest that progress will require experimental designs that integrate individual-level genomic data with deep, participant-informed deep phenotyping, capturing the combined effects of rare and common variants and environmental modifiers on disease expression and progression. We believe that an individualized precision medicine framework will uncover molecular drivers and modifiers of ME/CFS previously obscured by heterogeneity, enabling biologically informed stratification, improved trial design, biomarker discovery, and targeted interventions in this historically neglected condition.

**Supplementary Information:**

The online version contains supplementary material available at 10.1186/s12967-025-07586-w.

## Introduction

### Overview of ME/CFS

Myalgic encephalomyelitis/chronic fatigue syndrome (ME/CFS) is a complex, disabling, and frequently underdiagnosed and misdiagnosed disease. ME/CFS is characterized by disabling fatigue, post-exertional malaise (PEM), unrefreshing sleep, cognitive dysfunction, together with a range of other neurological, immune, and autonomic symptoms [1–3]. PEM, characterized by a delayed worsening of physical and cognitive symptoms after even minor exertion that was previously well tolerated, typically begins 12 to 48 hours after the trigger, can last for days or weeks, and is often accompanied by profound fatigue [1–3]. Symptoms vary in combination and severity, often fluctuating [1–3]. Precipitating events include infection, stress, and trauma, though many report no initiating event [1–3]. Diagnostic criteria such as the Fukuda, Canadian Consensus (Carruthers), and International Consensus Criteria define the condition based on hallmark features. The prevalence of ME/CFS has risen in the wake of the SARS-CoV-2 pandemic, with around half of Long COVID patients meeting ME/CFS diagnostic criteria, and over 1% of adults now affected with this syndrome (1.7% of women, 0.9% of men) [1, 2, 4–6]. The public healthcare crisis this represents has amplified the urgency to define ME/CFS etiology and develop frameworks for diagnosis and treatment. In 2023 ME/CFS was estimated to cost the U.S. economy $18–$51 billion annually [5].

## Current gaps

No definitive cause or diagnostic test for ME/CFS exists; diagnosis is based on exclusion and clinical assessment of symptoms, making it a lengthy and costly process not always attempted or completed [1–3, 5]. ME/CFS has no standardized treatment and no cure, care is typically prolonged trial-and-error with drugs (analgesics, antidepressants, beta-blockers, anti-inflammatories, antivirals) plus supplements and lifestyle changes [1–3, 5]. Previous studies have suggested immunologic, neurologic, mitochondrial, and metabolic mechanisms of disease [7–10], but no consistent subgroupings or biomarkers have been identified [11, 12]. Some twin, pedigree, or association studies report significant genetic risk [11, 13, 14], whilst others have reported little or none, and reproducible associations remain elusive [12, 14].

## A precision medicine approach

Considering prior genetic studies and leveraging our expertise in rare diseases, we hypothesized (as have others [7, 15]) that ME/CFS may have a significant genetic component characterized by substantial heterogeneity, in which distinct disorders with overlapping and convergent symptoms are grouped under a single clinical diagnosis. We further considered that this heterogeneity, amplified by differences in inclusion and exclusion criteria, diagnostic definitions, and recruitment strategies, may have led earlier studies to capture genetically distinct subgroups, leading to inconsistent findings, poor reproducibility, and dilution of disease-relevant signals in population-level analyses. We reasoned that failure to account for such genetic complexity would obscure signals and meaningful associations. A precision medicine framework, we hypothesized, might reveal individual-level molecular contributors that would otherwise remain hidden.

We applied a precision medicine framework integrating whole-genome and transcriptomic data in an individualized “n-of-1” design, incorporating participant-informed phenotyping, machine-learning–guided variant prioritization, transcript-aware pathogenicity prediction, and expert curation. We identified likely molecular contributors in 13 families, with impacted loci mapping to biologically plausible pathways, offering hypothesis-generating insights consistent with previously reported disease mechanisms and observed symptoms. Even within this small cohort, emerging molecular patterns suggestive of the existence of subgroups, laying the groundwork for identification of targeted interventions, biomarker discovery, and more precise clinical trial design.

## Materials and methods

### Cohort recruitment

Participants were recruited from the Birmingham, Alabama area under IRB approval from the University of Alabama at Birmingham (IRB-300000911; Genetic Testing in Chronic Fatigue Syndrome; open for recruitment from 15 August 2018 to present). Written informed consent was obtained for genomic analysis and de-identified symptom data sharing. Structured clinical assessments were used to ensure diagnostic consistency across the cohort. Initial eligibility was assessed via phone screening covering symptoms, disease severity, comorbidities, and medications. Screening included review of physician records, confirmation of diagnosis, exclusion of alternative medical explanations, and application of exclusionary criteria (Supplemental Table 1). Those meeting criteria were invited for in-person screening at the UAB Clinical Research Unit, where final eligibility was confirmed. A ME/CFS focused clinical questionnaire (Supplemental File I) was used to gather detailed information on symptom history, comorbid diagnoses, medications, onset pattern, socioeconomic status, and disease trajectory.

**Table 1:** Prioritized molecular variants identified by participant. This table summarizes likely or confirmed pathogenic molecular findings in 17 affected individuals from 13 families. Those shown have definitive variants per our tiering. Each entry lists the participant id, disease severity, gene(**s**) involved, variant(**s**) identified, variant-level scores (RVIS, CADD, DITTO), zygosity, ACMG classification, and confidence level. Functional information, tissue expression, associated OMIM curated conditions, and relevant clinical features are also provided. Variant classifications follow ACMG/AMP guidelines, incorporating expert manual review, segregation data, in silico predictions, and literature or ClinVar support. Gene functions and tissue expression were retrieved from Ensembl and GTEx, and associated conditions were derived from OMIM and/or ClinGen. Participants are grouped into molecular categories based on shared biological processes: SOL (solute and ion transport), ENE (energy metabolism and mitochondrial function), RBC (red blood cell structure and metabolism), and STR (structural integrity and muscle function). Severity reflects burden derived from structured interviews and questionnaires. References correspond to primary literature supporting gene–disease associations

Participant	Severity	Gene(s)	Variant	RVIS, CADD, DITTO	Zygosity, ACMG Class, Confidence	Gene Function	Expression	Associated Conditions (OMIM ID)	Clinical Features (ClinGen Clinical Actionability)*	References	Grouping
1	Moderate	KCNJ18	p.Gln407Ter	RVIS = NA CADD = 36.0 DITTO = 1.0	Homozygous, Pathogenic, Definitive	Inward rectifier potassium channel; maintains resting membrane potential, stabilizes muscle and nerve excitability.	Skin, Bone marrow and Lymphoid tissues	Muscle channelopathies, Thyrotoxic Periodic Paralysis. (613239)	Episodic muscle weakness, cramps, flaccid paralysis, fatigue, exertion intolerance, and sleep disturbances. Symptoms triggered by stress, cold, exertion, or carb-rich meals. (Not curated)	72,73	SOL
2	Moderate	KCNJ18	p.Leu156Pro; p.Thr354Met; in cis	RVIS = NA CADD = 17.95 DITTO = 0.05; CADD = 24.2 DITTO = 0.03	Heterozygous, Pathogenic, Definitive; Heterozygous, VUS, Suggestive						SOL
3	Moderate	KCNJ18	p.Arg399Ter; p.Ser314Asn	RVIS = NA CADD = 33.0 DITTO = 1.0; CADD = 25.7 DITTO = 0.06	Heterozygous, Pathogenic, Definitive; Heterozygous, VUS, Suggestive						
4	Moderate	KCNJ18	p.Arg399Ter	RVIS = NA CADD = 33.0 DITTO = 1.0	Heterozygous, Pathogenic, Definitive						
19, 20	Moderate/Severe	SLC12A3	p.Val270GlyfsTer39	RVIS = −1.62 CADD = 34 DITTO = NA	Heterozygous, Pathogenic, Definitive	Sodium-chloride cotransporter; regulates electrolyte balance and supports neuromuscular function and fluid homeostasis.	Kidney, Endometrium, Adipose, Blood, Immune	Gitelman Syndrome, Secondary electrolyte disturbance syndromes. (263800)	Chronic fatigue, muscle cramps, weakness, and frequent urination with metabolic alkalosis due to hypokalemia and hypomagnesemia. Symptoms may worsen during stress or illness. (Not curated)	78,79	SOL
5	Moderate	ENO3	p.Gly348ArgfsTer5	RVIS = −0.49 CADD = 35 DITTO = NA	Heterozygous, Pathogenic, Well supported	Skeletal muscle glycolytic enzyme; enables ATP production during anaerobic exertion and supports muscle energy needs.	Skeletal muscle, Heart	Glycogen storage disease XIII. (612932)	Exercise-induced fatigue, muscle pain, cramps, and post-exertional weakness. Severe cases involve rhabdomyolysis or myoglobinuria. Triggered by high energy demand. (Not curated)	39,40	ENE
7, 8	Moderate/Severe	ACADM	p.Leu84Phe	RVIS = −0.25 CADD = 25.6 DITTO = 1.0	Heterozygous, Pathogenic, Well supported	Mitochondrial enzyme; catalyzes medium-chain fatty acid oxidation for energy during fasting, illness, and stress.	Kidney, Heart, Liver, Muscle, Epithelial, Skin, Adipose	Medium-chain acyl-coenzyme A dehydrogenase deficiency, Metabolic stress-related myopathy. (201450)	Fatigue, cognitive issues, weakness, and exercise intolerance in late-onset cases. Early forms; seizures, hypoglycemia, and hepatomegaly, triggered by illness or exertion. (ClinGen: Strongly actionable)	43,44	ENE
15	Moderate	HADHA	p.Glu510Gln	RVIS = −0.64 CADD = 28.8 DITTO = 0.996	Heterozygous, Pathogenic, Well supported	Trifunctional mitochondrial enzyme complex; mediates long-chain fatty acid oxidation for energy during fasting, illness, and stress.	Heart, Muscle, Epithelial, Liver, Skin, Small intestine, Colon, Kidney, Blood, Immune	Long-chain 3-hydroxyacyl-CoA dehydrogenase deficiency, Maternal HELLP syndrome, AFLP. (609016, 609,015)	Fatigue, pain, neuropathy, hypoglycemia, and rhabdomyolysis, with onset during fasting, illness, of physical exertion. Presentation ranges from early severe to milder adult forms. (ClinGen: Moderately actionable)	50,51	ENE
18	Moderate/Severe	MMACHC	p.Arg91LysfsTer14	RVIS = −0.13 CADD = 24.4 DITTO = 1.0	Heterozygous, Pathogenic, Well supported	Vitamin B12- processing enzyme; supports mitochondrial energy metabolism, methylation, and neurological stability.	Esophagus, Liver, Skin, Blood, Immune, Epithelial, Endothelial, Muscle	Vitamin B12 metabolic defect, Combined methylmalonic acidemia and homocystinuria. (277400)	Fatigue, cognitive dysfunction, weakness, psychiatric symptoms, and anemia in later-onset forms. Early-onset may involve seizures, hypotonia, proteinuria, and DDID. (Not curated)	63,64	ENE
		ELOVL4	p.Thr233Met	RVIS = 0.39 CADD = 26.7 DITTO = 0.999	Heterozygous, Likely Pathogenic, Suggestive	ER-bound Carbon transfer enzyme; first step in synthesis of long-chain fatty acids, responsible for production of very long-chain fatty acids.	Eye, Esophagus, Liver, Skin, Blood, Immune, Epithelial, Endothelial, Muscle	Spinocerebellar ataxia 34, Spastic quadriplegia ichthyosis, Stargardt disease 3. (133190, 614,457)	Progressive nystagmus, bladder dysfunction, fasciculations, ataxia, dysarthria, hyporeflexia, peripheral neuropathy, progressive vision loss, and ichthyosis.(Not curated)	69,70	
21, 22, 23	Moderate/Severe	SPTA1	c0.4339-99C > T	RVIS = 4.85 CADD = 1.368 DITTO = 0.002	Heterozygous, Pathogenic, Definitive	Red blood cell (RBC) membrane alpha spectrin; maintains red blood cell shape, flexibility, and microvascular deformability.	Blood, Immune, Spleen, Liver	Hereditary Elliptocytosis, Hereditary Spherocytosis, Pyropoikilocytosis, Hereditary anemias. (130600, 266,140,270,970)	Early severe forms: neonatal anemia, jaundice, and growth delay. Milder: fatigue, hemolytic anemia, splenomegaly, and risk of aplastic crises following infection. (Not curated)	85,86	RBC
26	Severe	SPTA1	c0.4339-99C > T	RVIS = 4.85 CADD = 1.368 DITTO = 0.002	Heterozygous, Pathogenic, Definitive						RBC
		GMPPB	p.Asp27His	RVIS = −0.34 CADD = 24 DITTO = 0.999	Heterozygous, Pathogenic, Suggestive	Maintains glycosylation of alpha-dystroglycan, essential for muscle membrane stability and neuronal function.	Blood, Immune, Lung, Colon, Pancreas, Adrenal, Liver, Kidney, Muscle	Muscular Dystrophy Dystroglycanopathies. (615350, 615,351, 615,352)	Early forms: congenital muscular dystrophy with brain and eye anomalies, sometimes DDID. Later forms: limb-girdle weakness, hypotonia, exercise intolerance, fatigue, and muscle pain, often worsened by exertion or infection. (Not curated)	95,96	RBC
24	Severe	SLC4A1	p.Gly448Val	RVIS = −0.79 CADD = 34 DITTO = 1.0	Heterozygous, VUS, Likely	RBC membrane chloride-bicarbonate exchanger; maintains red blood cell shape and stability, carbon dioxide transport, and acid-base balance.	Liver, Adrenal, Kidney, Spleen, Colon, Lung, Pituitary, Heart	Spherocytosis, Hereditary anemia, Ovalocytosis, Cryohydrocytosis, Distal renal tubular acidosis. (185020, 179,800, 611,590, 612,653)	Early severe forms: neonatal anemia, jaundice, metabolic acidosis, growth delay, and FTT. Milder forms: fatigue, muscle weakness, hemolytic anemia, jaundice, splenomegaly, kidney stones. (Not curated)	102,103	RBC
25	Moderate/Severe	MYH7	p.Asp906Gly	RVIS = −3.64 CADD = 24.7 DITTO = 1.0	Heterozygous, Pathogenic, Definitive	Myosin heavy-chain in cardiac and skeletal muscle; drives contraction and supports force generation during movement.	Heart, Muscle	Dilated and Hypertrophic cardiomyopathies, Congenital myopathies 7A and 7B. (613426, 608,358, 255,160, 160,500)	Muscle fatigue, weakness, dizziness, exertion intolerance, and autonomic symptoms, sometimes with cardiomyopathy. Symptoms may fluctuate with illness or physical activity. (Under curation)	104,105	STR

Inclusion criteria required a confirmed diagnosis of ME/CFS meeting the Fukuda 1994 definition [16], with additional cutoffs for moderate fatigue severity and frequency proposed per Jason and colleagues (2014) to reduce misclassification [15]. Whilst not used for inclusion/exclusion criteria, all participants also met the Canadian Consensus Criteria (CCC) criteria [17]. Primary symptoms must have been present for at least 6 months. Fatigue severity and frequency were assessed using the DePaul Symptom Questionnaire, a standardized and widely used self-report instrument specifically developed for ME/CFS characterization [18]. While these methods enable structured evaluation of symptom presence, frequency, and severity, reliance on retrospective recall may introduce variability or recall bias. Disease severity was further quantified using a single self-report item from the Brief Fatigue Inventory [19], which measures fatigue on a 0–10 scale (0 = no fatigue; 10 = fatigue as bad as imaginable); all participants reported at least moderate fatigue of severity ≥ 4.

Participants were limited to individuals between 18 and 70 years of age, with one individual enrolled at age 70, but 71 at the time of sample collection. Recruitment was restricted to females due to both the female predominance of ME/CFS and practical challenges in enrolling males; one affected male was included as part of an affected mother–son pair. While this design may introduce potential selection bias and limit generalizability to males, it reduces other sources of variability such as hormonal and sex-specific immune effects. Race and ethnicity were self-reported using NIH/OMB categories at enrollment (separate ethnicity and race checkboxes) and also derived from WGS data.

**Fig. 1:**
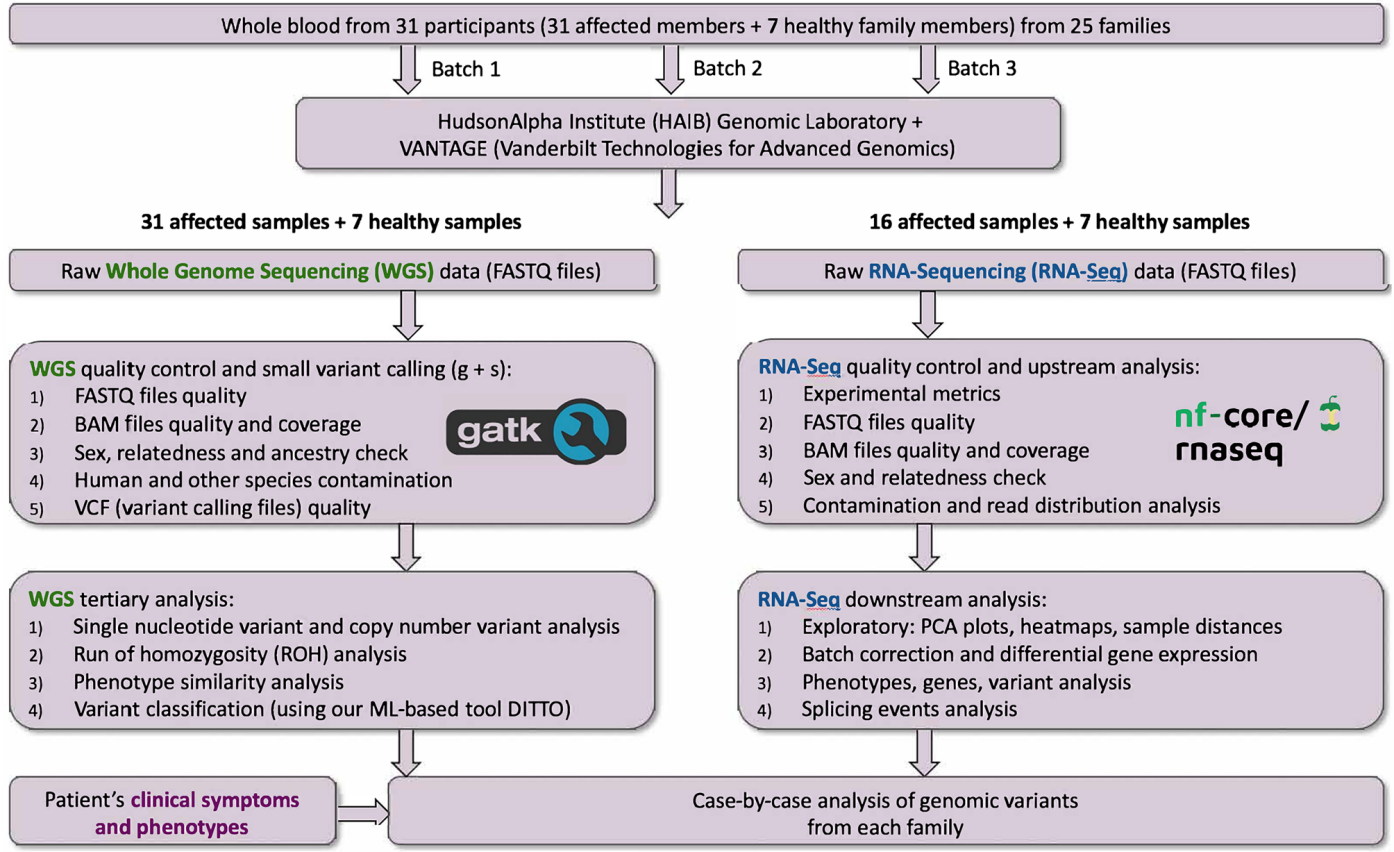
Experimental design, methods, and approach. Methods and design shown for whole genome sequencing (WGS) and bulk rna sequencing (RNA-Seq) components. WGS was performed at HudsonAlpha or the Vanderbilt Vantage lab to clinical-grade 30X coverage to generate paired-end 150-bp reads on HiSeqX or NovaSeq 6000 platforms. rna was isolated and post-isolation QC of total rna at the vantage lab was performed with integrity assessment. Library prep and sequencing was performed on an Illumina NovaSeq 6000, generating paired-end 150 bp reads targeting 100 million reads per sample

Exclusionary criteria included presence of rheumatological or autoimmune disorders with the exception of well controlled thyroid disease. Major depressive disorder, acute infections, pregnancy, and positive lab tests indicative of autoimmune disease were also exclusionary. Symptoms also had to be unexplained by other medical conditions based on history, examination, and diagnostic testing. Given the study’s molecular focus, affected and unaffected first-degree relatives were enrolled when available. Unaffected relatives were required to be same age or older than the affected participant and confirmed asymptomatic and free of ME/CFS symptoms by screening interview and questionnaire. Affected relatives met all ME/CFS inclusion criteria. The experimental design is shown in Fig. 1.

## Sample collection

Blood was collected for DNA and RNA analyses using PAXgene DNA and RNA tubes. Whole-blood provides a practical and standardized biospecimen that is easy to collect and process, facilitating participant compliance and reproducibility. However, for RNA-Seq it may miss disease-relevant expression changes due to the low abundance of affected tissues or cell types, variability in leukocyte composition, and transient inflammatory states that can influence gene expression profiles.

## DNA sequencing and analysis

DNA was isolated, quantified for yield and quality, and sequenced at either HudsonAlpha or Vanderbilt VANTAGE laboratories. Whole-genome sequencing (WGS) was performed to clinical-grade depth (≥30× mean coverage) using Illumina kits and flow cells (on HiSeqX or NovaSeq 6000 platforms) to generate paired-end 150 bp reads. Paired FASTQ files were aligned to the GRCh38 reference genome using BWA-MEM, and small variants were called with HaplotypeCaller following GATK Best Practices [20, 21]. Quality control of reads, alignments, and variant calls was performed using QuaC (v1.0) with default thresholds [22]. Median coverage ranged from 31× to 68× (Supplemental Table 2). Structural variants (SVs) were called using Manta (v1.6.0) [23], and regions of homozygosity (ROH) were identified with Automap (v1) [24] using a minimum ROH size of 0.5 Mb. SVs and ROHs were annotated and visualized using AnnotSV (v3.4), KnotAnnotSV (v1.1.4), and Samplot (v1.1.0) [25–27]. B-allele frequency aberrations were assessed using mixoviz (ver 2f2798a) [28]. Somalier [29] was used to extract informative sites, evaluate relatedness, and make ancestry predictions.

## Variant classification

Called variants were annotated and analyzed using previously described strategies [21, 30, 31]. In brief, pathogenicity was assessed by expert manual review following ACMG/AMP guidelines, integrating segregation data, population frequency, in silico predictions (including CADD [32], SpliceAI [33], DITTO [31]), and phenotype concordance. DITTO, our transcript-aware, explainable neural network, integrates population, conservation, splicing, UTR, gene constraint, and phenotypic features to generate per-transcript pathogenicity probabilities across all variant consequences and provides SHAP-based interpretability outputs [31]. DITTO shows improved accuracy for transcript-dependent and non-missense classes compared with conventional tools [31]. DITTO outputs were used to (i) resolve transcript discordance by prioritizing high-probability variants in disease-relevant, expressed transcripts (e.g. blood, PBMC, brain, or muscle) and (ii) scale ACMG PP3/BP4 strength to probability thresholds (≥0.90 = strong PP3; ≤0.10 = strong BP4), consistent with guidance [31]. Prioritized variants were reviewed by expert analysts and classified as pathogenic, likely pathogenic, VUS, likely benign, or benign per ACMG guidelines (Supplemental Table 3) [34]. All pathogenic variants were previously reported in ClinVar. All likely pathogenic variants met ACMG criteria based on frequency, inheritance, genotype-phenotype concordance, and functional and/or computational support.

Alongside ACMG classification, we applied a complementary four-tier confidence framework to evaluate each variant’s relevance to the patient’s clinical presentation. This system integrates information about the expected mode of inheritance, observed zygosity, and degree of phenotypic match to stratify findings as: (1) **Definitive** – a known pathogenic variant in a dominant gene fully consistent with the phenotype; (2) **Supported** – either a pathogenic or likely pathogenic heterozygous variant in a recessive gene or a likely pathogenic variant in a dominant gene with strong phenotypic overlap; (3) **Candidate** – a variant of uncertain significance with partial phenotypic match and/or transcriptomic support; and (4) **Uncertain** – a finding of interest lacking sufficient evidence at present but potentially relevant to future investigations.

Variants were also used to assign participants to molecularly defined subtypes based on the primary physiological system affected: Energy Production and Metabolism (ENE), Muscle Structure and Stability (STR), Red Blood Cell Membrane Integrity (RBC), and Solute Transport and Ion Homeostasis (SOL); participants with inconclusive findings were classified as Uncertain (UNC).

## RNA sequencing and analysis

RNA-seq was used to assess differential gene expression. RNA was isolated from blood and quality assessed at the VANTAGE lab (RIN = 7.6–10.0; mean = 9.0 ± 0.7). Libraries were prepared and sequenced on an Illumina NovaSeq 6000, generating paired-end 150 bp reads targeting ~100 million reads per sample. FASTQ files were processed with nf-core/rnaseq pipeline (v3.9), which incorporates FastQC [35] for quality control, followed by adapter trimming and filtering using TrimGalore [36]. Reads were aligned to the GRch38 reference genome using STAR [37] with transcript quantification using Salmon [38].

Differential gene expression analysis between affected and healthy participants was performed in R (v4.5.1) using DESeq2 [39] (v1.48.2), with condition and batch included as covariates. Batch correction was applied using limma (v3.64.1) [40]. Genes with adjusted *P*-value < 0.05 and absolute log2 fold change > 1 were considered differentially expressed. Hierarchical clustering of DEGs was performed as described previously [41], and heatmaps were generated using ComplexHeatmap [42] (v2.24.1). Gene annotations were retrieved with biomaRt [43] (v2.64.0), and expression plots for individual genes produced with the plotCounts function in DESeq2. Data handling used tidyverse [44] (v2.0.0).

Principal component analysis (PCA) was performed on variance-stabilized counts from DESeq2 to evaluate sample structure, batch effects, and global transcriptional structure. PCA was conducted in R and the first two principal components (PC1 = 30% variance; PC2 = 11%) were visualized with ggplot2 (v3.5.2). Samples were annotated by batch, sex, affected status, and molecular category. No distinct clustering by affected status, sex, or category was observed, indicating that biological grouping did not dominate global variance and that batch correction effectively minimized technical effects (Supplemental Fig. 1).

Because RNA-seq was performed on whole blood, genes not actively transcribed in leukocytes may be underrepresented. To evaluate potential confounding from leukocyte composition, immune cell–type deconvolution was performed using CIBERSORTx [45] with the LM22 leukocyte signature matrix (B-mode batch correction, 1000 permutations, quantile normalization disabled) [46]. Group-wise comparisons across “Affected” status and molecular “Category” were conducted using rstatix [47] (v0.7.3) for Mann–Whitney U and Kruskal–Wallis tests with Benjamini–Hochberg correction, MASS [48] (v7.3–65) for ordered logistic regression, pheatmap for correlation and Z-score visualization, and FSA (v0.10.0) for Dunn’s post hoc testing [49].

## Phenotype analysis

In order to study phenotype across participants, we extracted participant symptoms from participant notes, structured interviews, and questionnaires and mapped these to HPO terms [50, 51]. We generated a binary matrix of terms that we visualized as a clustered heatmap, grouping terms and participants and annotating columns by molecularly defined category [44].

**Fig. 2:**
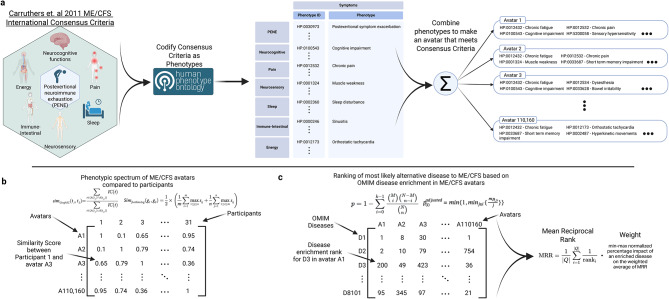
Generation of phenotypic avatars of ME/CFS using human phenotype Ontology (HPO) terms to support quantifiable similarity comparison. **a**) using the ME/CFS phenotypes in Supplemental Table 4 we produced all possible combinations of phenotypes that would minimally meet the Carruthers et. al 2011 ME/CFS diagnostic criteria. **b**) participant phenotypes were used to calculate their similarity to all 110,160 ME/CFS avatars. **c**) to determine which diseases shared phenotypic overlap with ME/CFS we performed OMIM curated disease enrichment analysis for each avatar, computed the mean reciprocal rank (MRR) for each disease, and weighted the MRR on the percent impact on the weighted average

In order to (i) test our hypothesis about the permissive nature of ME/CFS diagnostic criteria, (ii) determine whether each of our participants had representative ME/CFS presentations, and (iii) create a reference set of ME/CFS phenotype “avatars” for downstream comparisons, we converted the Carruthers et al. ME/CFS diagnostic criteria [2] into HPO terms (Supplemental Table 4) and enumerated all mathematically valid symptom combinations meeting the minimum diagnostic thresholds, generating computational “avatars” each meeting all diagnostic criteria (Fig. 2a).

To quantify similarity between participants and avatars, similarity was calculated across HPO terms using the hpo3 [51] Python/Rust library, which applies a graph-based information coefficient (HPOSim, eq. 7) to quantify similarity between individual terms and the “funSimAvg” method to aggregate similarity across full HPO profiles (Fig. 2b). To study enrichment in phenotypic similarity between (i) ME/CFS avatars and OMIM curated disease phenotypes and (ii) participants and OMIM curated disease phenotypes (Fig. 2c) we used a hypergeometric test implemented via the parallelized get_hypergeom_enrich function. A Benjamini–Hochberg (BH) correction was applied for multiple testing. Overall ranking for matches across all ME/CFS avatars was generated, accounting for both occurrence frequency and individual enrichment rank, using a weighted mean reciprocal rank approach.

## Results

### Participants

We enrolled 25 probands (38 individuals; spanning 31 affected participants and 7 healthy first-degree relatives). This included five affected-unaffected duos, three all-affected duos/trios, two trios with mixed unaffected/affected individuals, and 15 singletons. All affected individuals met Carruthers and CDC diagnostic criteria for ME/CFS. All probands and all but one affected individual were female; this male participant was included as an affected son in a mother–son duo. The mean age was 46.8 years (41.5 for affected participants, 71.7 for unaffected relatives). Race and ethnicity self-reported at enrollment agreed with that estimated from WGS data, showing a predominantly European cohort, with six individuals of substantial African ancestry and three with significant American admixture (Supplemental Figure 2). Disease onset ranged; eight in childhood, six in adolescence, nine in their twenties, and eight in their thirties. Sixteen reported gradual onset (four with identified exacerbating events), whilst 14 reported sudden onset: five with identified precipitating infections, six with non-infectious events, and three without any identified event. One participant did not complete the questionnaire but provided information via phone and in-person screening. All unaffected first-degree relatives were confirmed asymptomatic by interview and questionnaire. DNA and RNA were extracted and sequenced for all; due to sample storage issues at HudsonAlpha, samples were lost and RNA-seq was completed for 16 affected and 7 unaffected participants.

## Phenotypes

**Fig. 3:**
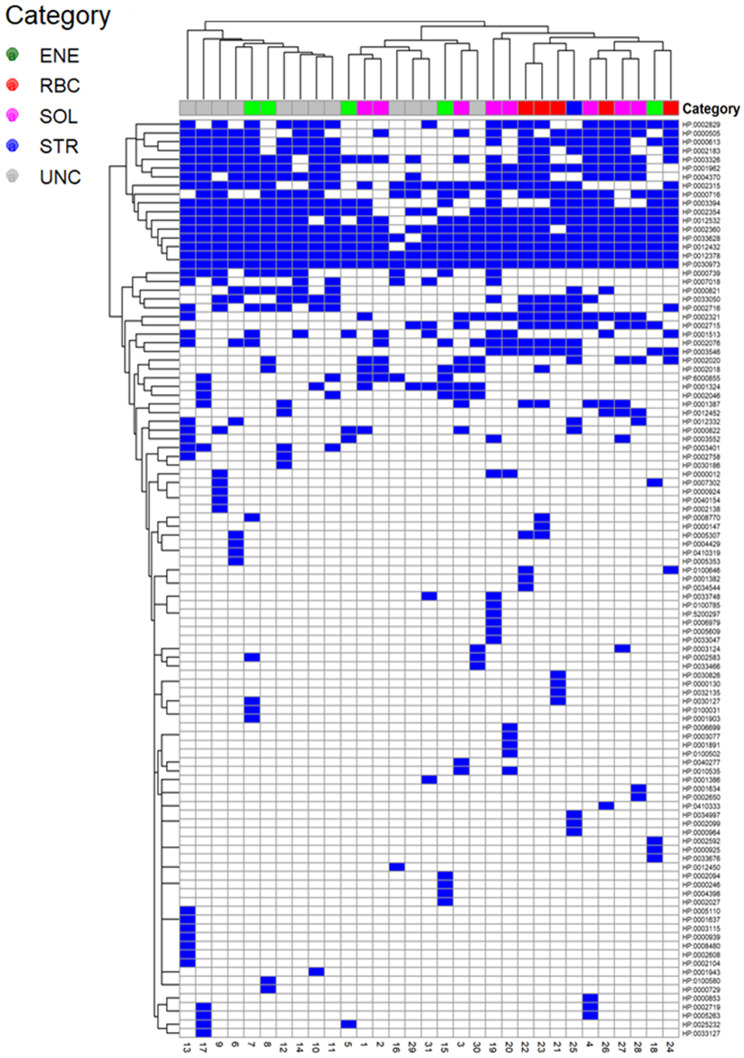
Participant dendrogram based on presence or absence of HPO terms across participants. Branch lengths represent topology only. Participant category is colored by molecular category, not phenotype. Affected individuals had between 9 and 37 HPO terms (on average 22). The heatmap shows distribution of HPO terms across participants, clustered by both HPO terms and participants. In some cases, clustering by family was noted, for example in mother and daughters 21, 22, and 23. Clustering by category was also noted, for example unrelated participants 1 and 2 who had known pathogenic KCNJ18 variants, and siblings 11–12 clustered with unrelated participant 14, all sharing the same rare CHRFAM7A splice VUS and symptoms (fatigue, pain, cognitive issues, and bowel irritability, but also restless legs, paresthesia, and other sensory, mood, and autonomic issues). Presumably due to small sample size, clusters were not statistically supported

As expected, symptom variation was seen across participants (Fig. 3). Fatigue and PEM were present in all individuals and chronic fatigue, pain, and sleep disturbance were common. Pain manifestations included muscle cramps, myalgia, arthralgia, joint pain, headaches, migraine, abdominal pain, sore throat, and facial pain. Autonomic symptoms, such as palpitations, vertigo, temperature intolerance, photophobia, phonophobia, and dysregulated thermoregulation were also relatively common, as were cognitive and mood-related symptoms (depression, anxiety, memory impairment, attention deficits) and gastrointestinal complaints (bowel irritability, nausea, reflux). In contrast, several features were seen in only a handful or single cases, including metabolic and hematologic abnormalities such as hepatic steatosis, hemolytic anemia, hypocalcemia, and coagulation defects, as well as neuromuscular manifestations including myopathy, ptosis, ataxia, peripheral neuropathy, and hypotonia.

Enumeration of all mathematically valid HPO-based symptom combinations meeting Carruthers ME/CFS criteria yielded 110,160 distinct diagnostic “avatars,” highlighting the breadth of phenotypic heterogeneity encompassed by current definitions. Similarity analysis across these avatars revealed substantial variability, with large numbers showing minimal overlap in symptom composition; particularly when core features were excluded (Supplemental Figure 3).

To evaluate whether ME/CFS diagnostic criteria capture symptom profiles characteristic of known genetic diseases, we tested for phenotypic enrichment of OMIM-curated Mendelian disorders within the ME/CFS avatar space. Despite the heterogeneity seen amongst the 110,160 avatars, enrichment analysis revealed statistically significant overlap with 135 OMIM-defined disorders after multiple-testing correction (Supplemental Table 5), indicating that a substantial subset of ME/CFS-compatible symptom constellations match those observed in established monogenic conditions. Enriched matches included disorders of thyroid hormone metabolism (THRA, THRB); mitochondrial DNA depletion and oxidative phosphorylation (SURF1, ACAD9, TACO1); fatty acid oxidation (HADHA, ACADM); excitation–contraction coupling and myopathies (CASQ1, RYR1, CACNA1S); renal ion handling, including Gitelman and hypomagnesemia syndromes (SLC12A3, CLDN16); congenital myasthenic syndromes (CHRNE, CHRND, COLQ, RAPSN); and autoinflammatory or immunodeficiency syndromes (NLRP12, MVK, NLRP3, PSMB8, ADA2).

Similar analyses performed between participant’s phenotype terms and OMIM-curated disease phenotypic profiles also revealed enrichment for mitochondrial and metabolic disorders, congenital myopathies, renal tubulopathies, and neurodevelopmental syndromes, and familial autoinflammatory disorders (Supplemental Table 6).

## Molecular findings

**Fig. 4:**
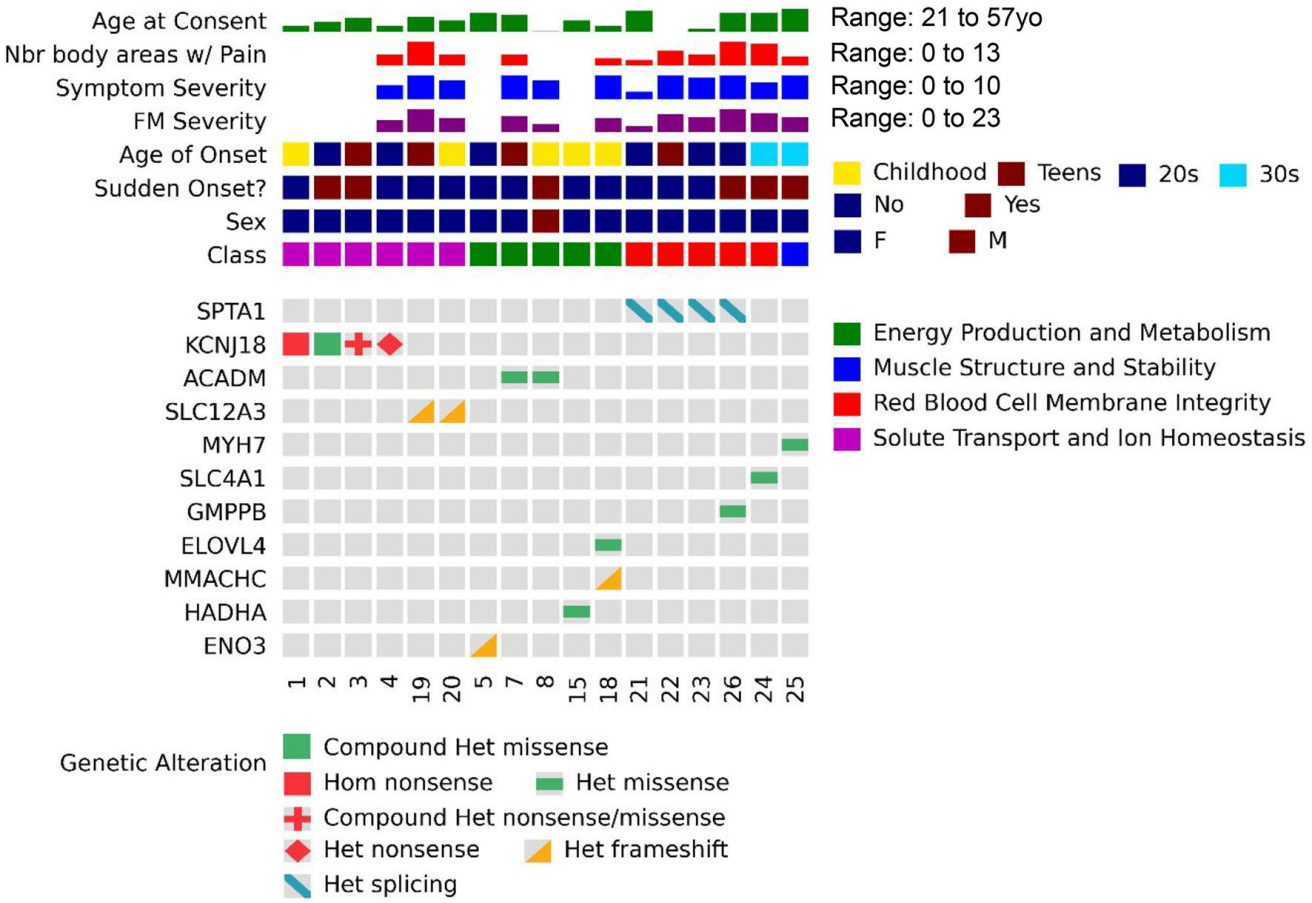
Heatmap summarizing demographic, clinical, and genetic findings across probands. The cases shown have definitive variants per our tiering. Top rows show age at consent, number of body areas with pain, symptom severity, fibromyalgia (FM) severity, and age of onset, with color scales indicating value ranges (right). Additional metadata include sudden onset (yes/no), sex, and molecular class. Molecular classes were assigned based on the primary physiological domain impacted by identified variants: energy production and metabolism (green), muscle structure and stability (blue), red blood cell membrane integrity (red), solute transport and ion homeostasis (magenta). Bottom panels show molecular findings. Columns = participants; rows = genes; shapes = zygosity and type of alteration; colors = impact. Symptom severity, pain, and fatigue scores were broadly similar across molecular categories, though trends suggested higher pain and overall severity in Red Blood Cell (RBC) and energy production (ENE) groups compared to solute transport (SOL). Participants with solute transport associated variants more often reported sudden onset, while RBC and metabolic groups tended toward earlier onset and greater symptom burden

We identified definitive findings in 8/25 probands (32%) and 12/31 (39%) of affected individuals (Table 1, Fig. 4). These variants met ACMG pathogenicity criteria, were classified as pathogenic in ClinVar or supported by strong literature, and mapped to dominant disorders whose phenotypes aligned with core ME/CFS symptoms and participantreported features. In four additional probands (five participants total), we identified heterozygous pathogenic variants in genes associated with recessive disorders; while not sufficient for full disease manifestation, symptom overlap suggests possible modifier or partial-effect roles. In another case, a heterozygous VUS in a dominantly inherited disease gene (SLC4A1) showed phenotypic concordance with the participant’s presentation and gene expression changes consistent with a deleterious effect (Supplemental Figure 4, Supplemental Table 3).

**Fig. 5:**
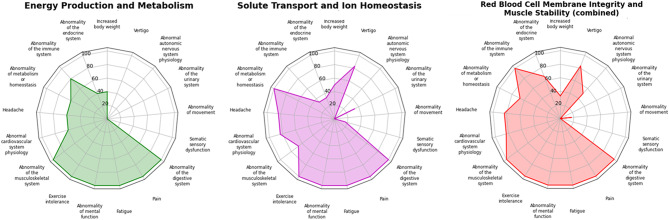
Participant symptom profiles from HPO terms grouped by functional class. Radar plots summarize findings by participant’s molecular findings; each spoke is an HPO term (collapsed to parent terms for sparsely reported, related items), and spoke length shows the percent of participants in that group affected for that HPO symptoms/category (0 at center, 100 at rim). ME/CFS-defining features (as translated to HPO terms); fatigue, pain, and exercise intolerance, as well as digestive abnormalities were seen in all individuals regardless of molecular subtype. Musculoskeletal, metabolic, cardiovascular findings and headaches were common. Solute transport cases showed higher numbers of metabolism/homeostasis symptoms and cardiovascular symptoms and vertigo with relatively fewer immune features. RBC/muscle stability cases more frequently had immune, cardiovascular, and autonomic issues. Energy & metabolism cases also more frequently showed immune findings, with fewer autonomic abnormalities

These variants affect genes expressed in cell types with key physiological roles that provide strong biological plausibility for contributing to canonical ME/CFS symptoms (Fig. 5, Supplemental Figure 5). Variants affecting energy production and metabolism (*ENO3, HADHA, ACADM, MMACHC*) disrupt mitochondrial function, glycolysis, and fatty acid oxidation, and are causally associated with PEM, chronic fatigue, muscle pain, weakness, and cognitive dysfunction. Variants in solute transport and ion homeostasis (*KCNJ18, SLC12A3*) impair electrolyte balance, neuronal signaling, and vascular tone, resulting in exercise-induced fatigue, muscle cramps and weakness, cognitive issues, and orthostatic intolerance. Variants in red blood cell membrane genes (SPTA1, SLC4A1) impair erythrocyte function, hemolysis, perfusion, microvascular flow and oxygen delivery, associated with chronic and exertion-induced fatigue, muscle pain, cognitive dysfunction, anemia, and pain. An *MYH7* variant impacting muscle structure and contractility causes activity intolerance, chronic fatigue, and myalgia resulting from impaired contraction, increased exertional strain, and reduced recovery capacity.

In several cases, RNA-seq provided orthogonal evidence for the predicted defect, revealing disease-relevant transcriptional changes. In others, it supported downstream consequences consistent with the proposed pathophysiology, identifying differentially expressed genes aligned with expected mechanisms (Supplemental Figure 4). As RNA-seq was performed on whole blood, the strongest mechanistic support was observed for variants in genes with expression in hematopoietic or immune cells.

Notably, the previously discussed enrichment analysis of combined participant HPO profiles against OMIM-curated disease phenotypes identified fourteen disorders that directly aligned with pathogenic or likely pathogenic variants seen in our cohort (Supplemental Table 6). These included thyrotoxic periodic paralysis (KCNJ18), fatty acid oxidation disorders (HADHA, ACADM), glycogen storage disease (ENO3), Gitelman syndrome (SLC12A3), cobalamin deficiency (MMACHC), erythrocyte and hemolytic disorders (SPTA1, SLC4A1, G6PD), and structural or metabolic myopathies (COL6A3, MYH7, GMPPB). This overlap reinforces the biological plausibility of the variants identified and their relevance to participant phenotypes.

Given the paucity of reported genetic findings in ME/CFS, the pronounced heterogeneity observed in our cohort, and the critical importance of sharing detailed molecular data to inform future research, we present detailed variant-level discussion, grouped by functional category (Table 1). Discussion of additional symptom–molecular associations are given in Supplemental File II.

## Energy production and metabolism

Participant 5 carried a pathogenic frameshift variant in *ENO3* (β-enolase), a glycolytic enzyme expressed in skeletal muscle and heart [52–54]. The variant disrupts dimerization, leading to monomerization, proteolysis, and reduced activity, to ~25% for ββ homodimers and ~50% for αβ heterodimers [52–54]. Loss of activity below ~50% causes Glycogen Storage Disease XIII, characterized by exertion-induced fatigue, weakness, pain, cramps, and rhabdomyolysis [54, 55]. Although ENO3 deficiency is recessive, heterozygotes can develop symptoms under stress or exertion [54, 55]. This participant’s prolonged post-exertional fatigue, weakness, pain, cramps, spasms, and tenderness are consistent with ENO3 dysfunction. Laboratory findings of low-normal phosphorus and mildly elevated bicarbonate may indicate mild or compensated metabolic alkalosis [54, 55].

Participants 7 and 8 (mother–son) shared a pathogenic *ACADM* missense variant, impairing medium-chain (C4–C12) fatty acid β-oxidation, causing medium-chain acyl-CoA dehydrogenase deficiency [56, 57]. Although classically recessive, heterozygotes can develop fatigue, weakness, and exercise intolerance, particularly during stress, illness, or fasting [57–60]. Both participants reported exertion-induced fatigue, cognitive dysfunction, and weakness after stressors, consistent with prior reports [57–60]. Participant 7 showed transcriptomic evidence of a metabolic shift, with increased PGM1 suggesting glycolytic compensation, reduced ACADVL suggestive of feedback inhibition or disrupted PPARα/PGC-1α signaling, and elevated NR3C1 reflecting glucocorticoid-driven metabolic regulation, changes not seen in Participant 8. Participant 7 also carried a pathogenic *COL4A3* variant, associated with basement membrane integrity in kidney, ear, and eye [61]. Both participants exhibited markedly elevated DEFA1 expression, consistent with neutrophil activation and innate immune system engagement [62].

Participant 15 carried a pathogenic *HADHA* variant affecting mitochondrial trifunctional protein, which catalyzes the final steps of long-chain fatty acid β-oxidation [63, 64]. Deficiency typically causes muscle weakness, fatigue, and exercise intolerance, particularly during stress, fasting, or infection [63, 64]. Although recessive, heterozygotes can manifest symptoms, with severity influenced by residual enzyme activity and physiologic stressors such as illness, fasting, or pregnancy; a pattern commonly seen in long-chain fatty acid oxidation disorders [65–68]. This participant’s fatigue, PEM, weakness, pain, muscle spasms, sensitivity, and nausea align with this finding. Additional VUSs in *NLRC4*, *SLC16A3*, and *SLC12A9* may contribute to inflammatory, muscle, and metabolic processes, if deleterious [69–75].

Participant 18 carried a pathogenic frameshift variant in *MMACHC* (p.Arg91LysfsTer14), which disrupts vitamin B12 processing and mitochondrial function, causing methylmalonic aciduria and homocystinuria type CblC [76, 77]. In a homozygous state, this variant causes severe neonatal-onset disease [76, 77], while milder, later-onset forms involving other variants present with neuropathy, fatigue, cognitive and psychiatric symptoms, incoordination, pain, GI issues, and weakness [76, 78–81]. No second variant was identified in this gene. A second, previously reported likely pathogenic variant was seen in ELOVL4 [82, 83], causal for a dominant slowly progressive very long-chain fatty acid elongation defect associated cerebellar ataxia (SCA34) presenting with ataxia and dysarthria, with or without dermatologic manifestations [82–84]. Interestingly, prior work has reported digenic disease involving presence of MMACHC and ZEB2, associated with reduced mitochondrial respiration and increased oxidative damage, particularly during fasting in skeletal muscle. Though intriguing, in the absence of functional evidence for a similar digenic interaction, this finding is purely speculative.

## Solute transport and Ion Homeostasis

Unrelated Participants 1–4 each carried known pathogenic variants in *KCNJ18*, encoding the Kir2.6 inward-rectifying potassium channel [85–87]. Participant 1 was homozygous, the remainder heterozygous. A single pathogenic variant is sufficient to cause dominant thyrotoxic periodic paralysis, characterized by disrupted muscle excitability and incomplete penetrance, with episodes triggered by thyrotoxicosis, fasting, stress, exertion, or carbohydrate loading [85–87]. Participants 1, 3, and 4 each had premature truncating variants. Their symptoms of muscle fatigue, cramping, PEM, episodic weakness, and exertion intolerance align with features of KCNJ18 channelopathy. Similar symptoms were seen in Participant 2, who carried T354M and L156P variants in cis; T354M abolishes Kir2.6 inhibitory response to activated Protein Kinase C signaling (elevated in thyrotoxicosis [86]), whilst L156P increases trafficking from the ER to the cell surface [88]. Participants 1 and 4 reported time of enrollment thyroid dysfunction, consistent with KCNJ18 disease mechanism. Emerging evidence suggests that variants in other ion channels may modify penetrance and phenotype in Kir-associated periodic paralysis [85, 86, 89]. Participant 4 also carried a *CACNA1S* VUS, a gene known to cause familial hypokalemic periodic paralysis, which could contribute to episodic weakness, if pathogenic [90].

Participants 19 and 20 (siblings) carried a likely pathogenic *SLC12A3* frameshift variant, the gene linked to Gitelman syndrome [91–93]. This hypokalemic metabolic alkalosis disorder leads to muscle weakness, cramps, pain, fatigue, hypovolemia, polyuria, and exercise intolerance during illness, fasting, or exertion [91–93]. Although classically recessive, heterozygotes can exhibit symptoms and dominant presentations have been reported [94, 95]. Both reported symptoms consistent with Gitelman syndrome, including muscle weakness, joint pain, cramps, chronic fatigue, severe overactive bladder (suggestive of polyuria), and exercise intolerance. Participant 19 also carried a pathogenic *COL6A3* variant, previously seen in dominant Bethlem myopathy 1A and Ullrich congenital muscular dystrophy 1A [96, 97], which may explain their greater pain, fatigue, and musculoskeletal involvement compared with their sibling.

## Red blood cell membrane integrity

Mother–daughter participants 21, 22, and 23, together with unrelated participant 26, carried a pathogenic splice *SPTA1* variant essential for erythrocyte flexibility [98, 99]. RNA-seq confirmed intron retention and truncation. Pathogenic *SPTA1* variants cause hereditary elliptocytosis, pyropoikilocytosis, spherocytosis, and hemolytic anemia, presenting with fatigue, PEM, dyspnea, reduced exercise tolerance, dizziness, and pain, often triggered by infection, exertion, or medication [98–100]. Symptoms arise at ~50% residual activity [101, 102]. All participants reported overlapping features consistent with this phenotypic spectrum.

Participants 21–23 also carried a pathogenic *F5* variant [103], and participants 22 and 23 an additional pathogenic *G6PD* variant [104], both of which may increase susceptibility to oxidative stress, hemolysis, and episodic fatigue, weakness, dizziness, and pain. All three shared a VUS in *CENPF*, a gene previously implicated in systemic lupus erythematosus [105–107]. Participant 26, who noted greater pain and broader symptoms also had a pathogenic *GMPPB* variant affecting α-dystroglycan glycosylation [108, 109]. Resulting dystroglycanopathies are typically early onset recessive, but can be later onset and milder with exercise intolerance, cramps, myalgia, fatigue, and progressive weakness, triggered by illness, pregnancy, or exertion [108–110]. This participant also had a pathogenic *CFTR* variant and VUSs in *HAGHL* and *FCRL1* [111–114].

Participant 24 carried a VUS in *SLC4A1*, encoding a chloride–bicarbonate exchanger critical for erythrocyte stability and renal acid–base balance [115, 116]. The variant lies adjacent to the anion exchange motif near pathogenic alleles causing dominant and recessive spherocytosis, ovalocytosis, cryohydrocytosis, and hemolytic anemia, characterized by hypokalemia, polyuria, weakness, and fatigue [115, 116]. RNA-seq showed elevated SLC4A1 expression and erythrocyte membrane gene (SPTA1, SPTB, EPB42, GYPA) dysregulation, suggestive of a compensatory response to red cell fragility or oxidative stress [98, 100–102]. This hypothesis-generating finding parallels expression changes in *SPTA1* carriers and warrants functional validation.

## Muscle structure and stability

Participant 25 carried a pathogenic *MYH7* variant affecting the cardiac myosin heavy chain [117]. *MYH7* variants cause dominant/recessive myopathies characterized by muscle pain, fatigability, weakness, dyspnea, dysautonomia, and late-onset cardiomyopathies [118, 119]. The participant’s symptoms; exercise intolerance, fatigue, dyspnea, dysautonomia, and neck pain fit within this spectrum [120–123]. A *MYH14* VUS, linked to peripheral neuropathy, myopathy, and hearing loss, was also identified and may contribute to myosin dysfunction in non-muscle tissues, if functional [124, 125].

## Other findings

Several participants harbored VUSs or likely pathogenic variants with phenotypic overlap with ME/CFS, but lacking sufficient evidence for definitive causality or inheritance patterns consistent with known disease mechanisms (Supplemental Table 3). These variants localize to physiological systems and molecular pathways previously implicated in ME/CFS pathophysiology, including immune regulation (*e.g. NLRP12*, *SIAE*) [126], which influences inflammatory signaling and immune homeostasis; energy metabolism (*e.g. TACO1*, *CUBN*, *TRPM2*), critical for mitochondrial function and nutrient processing; solute transport and neuromuscular excitability (*e.g. CHRNE*, *CACNA1B*, *CHRFAM7A*), which modulate ion gradients and neurotransmission; and red blood cell membrane integrity (*e.g. CD36*) [127], which affects oxygen transport and cellular resilience. While provisional, these variants are biologically plausible and, if confirmed, would expand ME/CFS’s molecular architecture and reveal mechanistic or therapeutic insights.

## Expression analyses

To study transcriptomic differences in ME/CFS participants, we first evaluated immune cell composition to rule out leukocyte subset variation as a potential confounder. Deconvolution analysis identified 22 immune subsets dominated by neutrophils, followed by monocytes and lymphocytes, consistent with expected peripheral blood distributions [128]. No significant differences were observed after multiple-testing correction (all adjusted *p* > 0.18), indicating stable immune-cell composition across samples and supporting that observed transcriptional differences were likely not driven by leukocyte subset variation.

We next examined gene-level expression changes. Participants with erythrocyte-related variants showed elevated expression of genes involved in red blood cell membrane integrity and erythroid stress responses (Supplemental Figure 4), suggesting compensatory upregulation. Some individuals with mitochondrial variants (e.g. *ACADM*) showed altered expression of glycolytic and mitochondrial regulatory genes. Upregulation of DEFA1, a neutrophil-derived antimicrobial peptide, was observed in some participants, aligning with prior reports of innate immune activation in post-infectious syndromes [62]. Expression changes were also seen in ZNF683, LRRN3, PIK3R3, CD248, and POLR3G suggested possible involvement of cytokine-driven T-cell regulatory networks. In particular, ZNF683 is linked to effector CD8^+^ and NK cell activation [129], while PIK3R3 is involved in TCR/IL-2/PI3K signaling [129, 130]. This immune signature partially overlapped with the immunoregulatory dysfunction regulatory network identified in ME/CFS by Hunter et al. [131].

## Genotype-phenotype concordance and disease enrichment

We re-examined phenotype-based clustering in light of molecular findings. Although clustering did not reach statistical significance; likely due to limited sample size, some grouping was seen (Fig. 3). For instance, siblings 11 and 12 and unrelated participant 14, all carrying a rare *CHRFAM7A* splice VUS, clustered with overlapping symptoms; pain, memory deficits, bowel irritability, sensory sensitivities, temperature dysregulation, mood changes, restless legs, stiffness, and paresthesia, consistent with potential α7 acetylcholine receptor dysfunction if the variant is deleterious.

Although avatar phenotypes were not used to guide WGS analysis, the disorders matched by avatar alignment aligned to our molecular findings, including gene overlaps and convergence across domains such as ion channel excitability, renal electrolyte handling, mitochondrial OXPHOS and mtDNA maintenance, fatty acid oxidation, glycolysis, lipid elongation, glycosylation, skeletal muscle function, and red blood cell function (Supplemental Table 5). Concordance also extended to our VUSs, which mapped to several avatar-enriched categories including OXPHOS/mitochondrial candidates, calcium-channel and synaptic loci, including genes implicated in hypokalemic periodic paralysis, solute and epithelial/channelopathy genes, and erythrocyte membrane/metabolic handling. Defects in genes associated with immune and barrier modulation, previously linked to ME/CFS and Long COVID [7, 8], were absent from pathogenic findings but observed among VUS (e.g. *NLRP12*, *NLRC4*, *SIAE*). In some cases, the HPO-based comparisons revealed phenotypic alignment with molecular findings in participants; for instance, Gitelman syndrome was the 4th most enriched match for Participants 19 and 20 (adj. *p* = 0.0084 and 7.9 × 10^− 8^), who had been found to harbor a pathogenic SLC12A3 variant causal for Gitelman’s.

## Discussion

Despite its prevalence and substantial impact on quality of life, ME/CFS remains both understudied and poorly understood. Genetic contributions, in particular, have received limited attention, in part due to early studies yielding inconsistent or non-reproducible findings, leaving the role of heritable variation in disease mechanisms and symptom expression underexplored.

This pilot study demonstrates the feasibility and potential impact of an n-of-1 precision medicine approach for ME/CFS by integrating whole-genome and transcriptome sequencing, machine learning–assisted expert prioritization, and participant-informed deep phenotyping. Using this n-of-1 strategy, we identified pathogenic variants with strong support in some participants and biologically plausible candidates in others.

Our results highlight the critical role of deep phenotyping, particularly when informed by detailed, participant-derived symptom histories. In a clinically heterogeneous disorder like ME/CFS; where diagnostic delays, prolonged diagnostic odysseys, and limited access to knowledgeable or receptive clinicians are common, early and/or defining symptoms are often missed or undocumented. Participant-provided narratives can reveal patterns, context, and clinical clues that standardized checklists or retrospective chart abstraction would miss, assisting with both genotype-phenotype correlation and interpretation.

Significant locus heterogeneity was observed in our cohort, with participants harboring variants in genes associated with mitochondrial and metabolic function (e.g. fatty acid oxidation, oxidative phosphorylation), skeletal and cardiac muscle (e.g. ion handling, contractility), central and peripheral nervous system function (e.g. autonomic regulation, neurotransmission), and red blood cell and vascular biology (e.g. oxygen transport). Several participants also carried candidate modifiers that may support generation of testable hypotheses about secondary disease contribution. Outwith our study, large-scale ME/CFS omics projects (e.g. DecodeME, HEAL [7, 132]) have implicated polygenic factors, some converging on the biological domains identified here presenting an additional avenue for analysis not performed here. Despite this complexity, many implicated pathways can be seen to converge on impaired energy production, reduced stress resilience, and vulnerability to post-exertional multisystem dysfunction; mechanisms consistent with the PEM, chronic fatigue, and cognitive impairment seen in ME/CFS (Supplemental File II).

Several findings suggest gene–environment interactions; for example, thyrotoxic periodic paralysis can be triggered by thyroid imbalance, exertion, infection, or fasting, while symptoms in fatty acid oxidation disorders often worsen during illness or fasting. These patterns underscore the need to capture symptoms, status, and context at the time of sample collection, as biomarkers may be missed if samples are taken outside periods of active disease or relevant exposures. More broadly, environmental and physiological stressors, including infections (e.g. EBV, SARS-CoV-2), surgery, toxin exposure, or trauma, may precipitate or amplify symptoms, onset, severity, and progression in genetically susceptible individuals, offering a mechanistic basis for relapsing–remitting patterns and interindividual variability. This heterogeneity and complexity, coupled with the permissiveness of ME/CFS diagnostic criteria, and varying inclusion and exclusion criteria, may help explain why prior studies, including larger GWAS, have yielded weak or irreproducible associations.

While genetic heterogeneity, compounded by diagnostic uncertainty, variable access to informed clinical care, and the influence of gene-gene or gene-environment influences pose challenges, heterogeneous findings may also provide opportunities for precision therapeutics. In a subset of participants, mechanistically interpretable variants suggest actionable strategies. Dietary modification may benefit those with fatty acid oxidation defects (e.g. *HADHA*, *ACADM*), thyroid dysfunction may be a modifiable driver in individuals with *KCNJ18* variation, and *SPTA1* variants point to potential roles for oxidative stress or folate metabolism. These individualized insights, while preliminary, suggest a rational path forward. Even when immediate treatment options are unavailable, defining a molecular etiology has clear value. It enables patient stratification, may inform prognosis by identifying more advanced cases with similar causes, and can facilitate inclusion in gene or pathway-specific registries and natural history studies; positioning individuals to benefit from future discoveries. Taken together, our findings establish the feasibility and clinical utility of an n-of-1 precision medicine framework for ME/CFS. By integrating genomics, transcriptomics, and deep patient-informed phenotyping, we uncovered plausible, testable molecular contributors in over a third of participants. While replication in larger, more diverse cohorts remains critical, the rarity of many variants identified suggests that population-scale validation for all may be impractical. Targeted recruitment based on phenotypic patterns or individualized approaches may offer a more effective path forward. The insights presented here provide a strong rationale for defining biologically anchored subtypes, guiding personalized interventions, and designing targeted clinical trials. Importantly, identifying a molecular basis, even in a subset of patients, can offer diagnostic clarity, reduce stigma, and provide patients and clinicians with a roadmap for future care. Moving forward, we feel that to refine mechanistic understanding, efforts may need to integrate deep, participant-informed phenotyping with assessments of rare variants, polygenic risk, and environmental exposures, while also accounting for the timing of sample collection. This approach reframes ME/CFS as a biologically diverse collection of disorders and opens new avenues for mechanism-based discovery in this long-neglected condition [133].

## Electronic supplementary material

Below is the link to the electronic supplementary material.

Supplementary Material 1

Supplementary Material 2

Supplementary Material 3

Supplementary Material 4

Supplementary Material 5

Supplementary Material 6

Supplementary Material 7

Supplementary Material 8

Supplementary Material 9

Supplementary Material 10

Supplementary Material 11

## Data Availability

Data from participants are subject to privacy/ethical restrictions and are available to verified researchers upon reasonable request by contacting the corresponding author. All the analysis scripts are available in the corresponding code repository (https://github.com/uab-cgds-worthey/mecfs-nof1).

## Declarations

### Code availability

The custom code used to perform the phenotypic and transcriptomic analyses in this study is released under the GNU General Public License v3 and is available at https://github.com/uab-cgds-worthey/mecfs-cohort-analysis. It is available at https://github.com/uab-cgds-worthey/mecfs-cohort-analysis (Zenodo DOI: http://doi.org/10.5281/zenodo0.17612946). The repository includes full documentation and versioned code sufficient for the reproduction of all computational results.
