# Supplementary figures and images for: Uncovering the genetic architecture of ME/CFS: a precision approach reveals impact of rare monogenic variation
Source: J Transl Med. 2025 Dec 24;24:168. doi: 10.1186/s12967-025-07586-w (PMC12888368; doi:10.1186/s12967-025-07586-w)

Principal Components Analysis

A

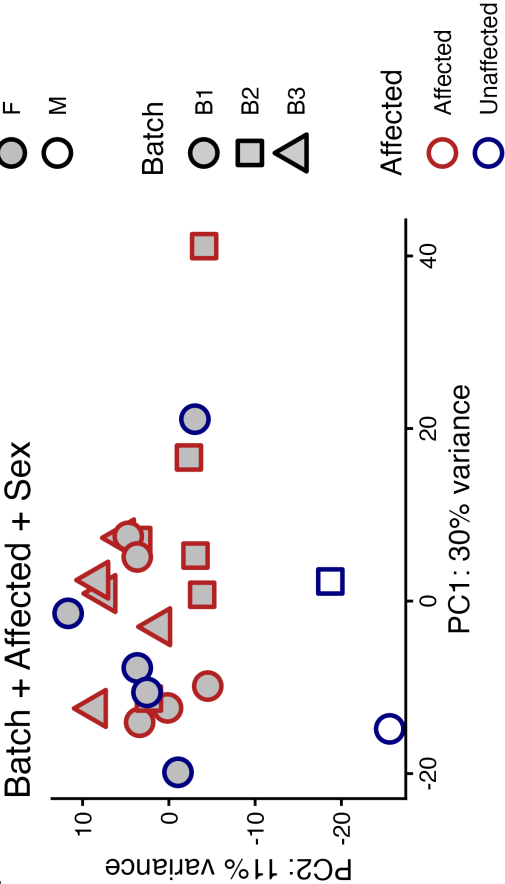

B

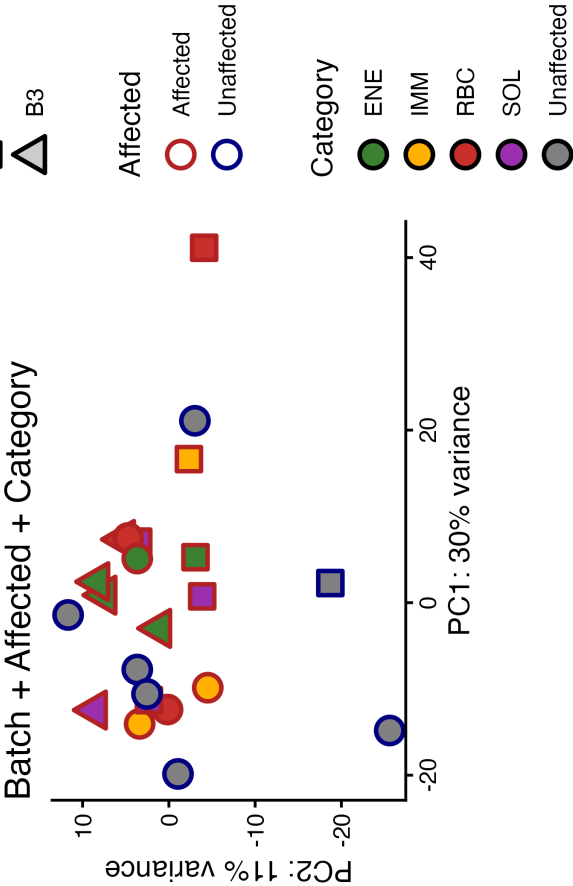

Supplement: Supplementary file 1 — Supplementary Material 1 [file 12967_2025_7586_MOESM1_ESM.pdf]

Predicted Ancestry Proportions

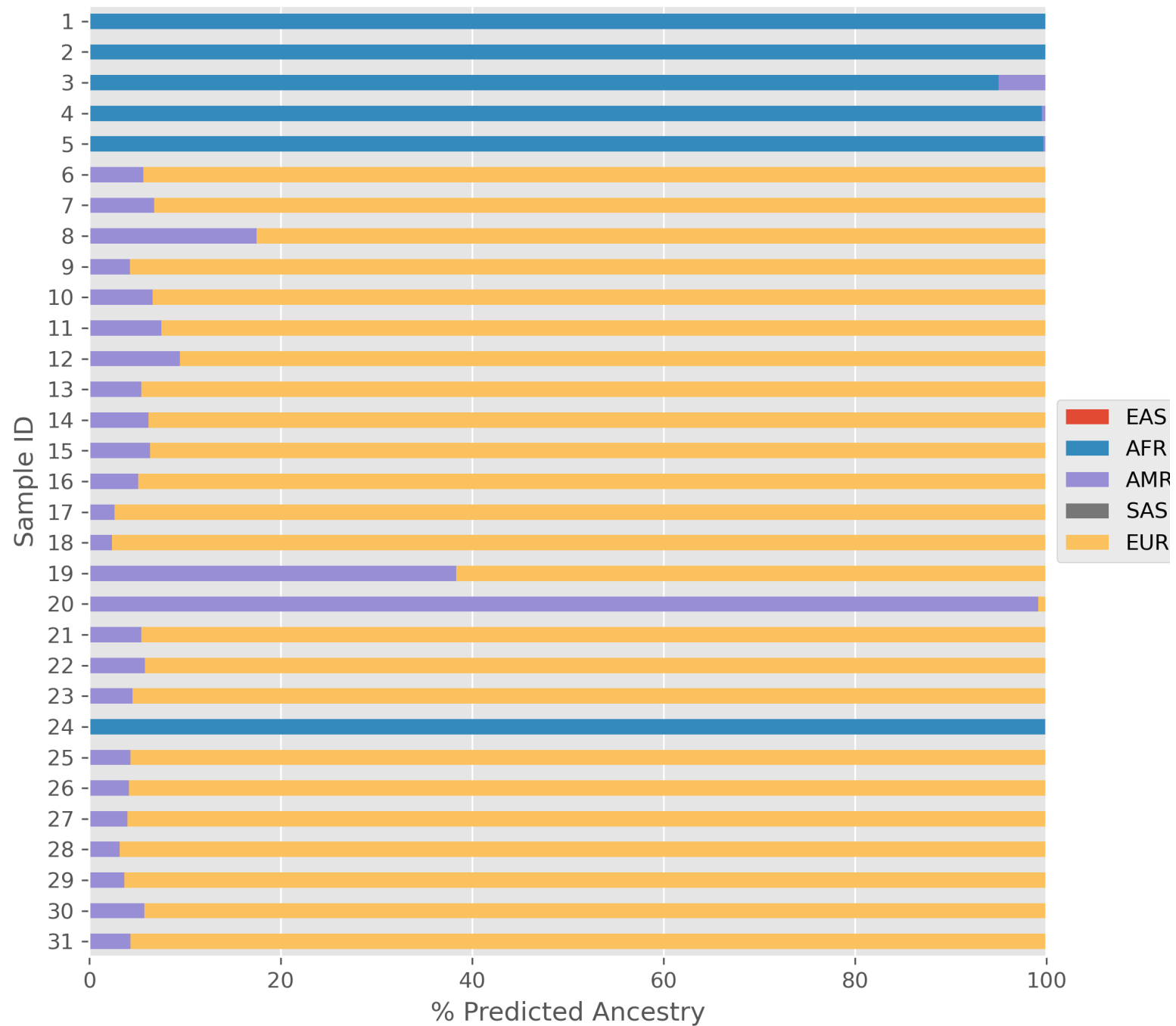

Supplement: Supplementary file 2 — Supplementary Material 2 [file 12967_2025_7586_MOESM2_ESM.pdf]

A.

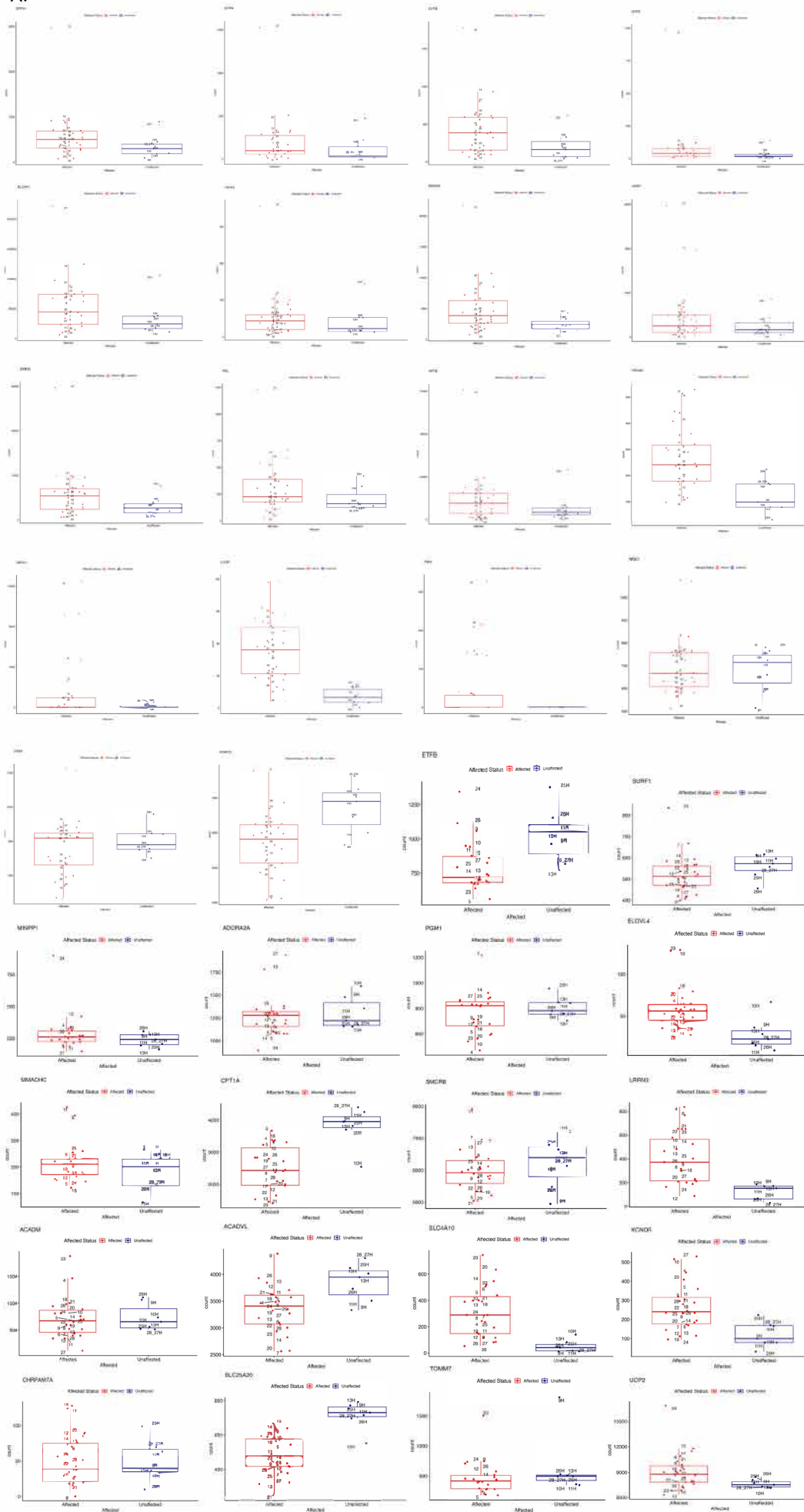

B.

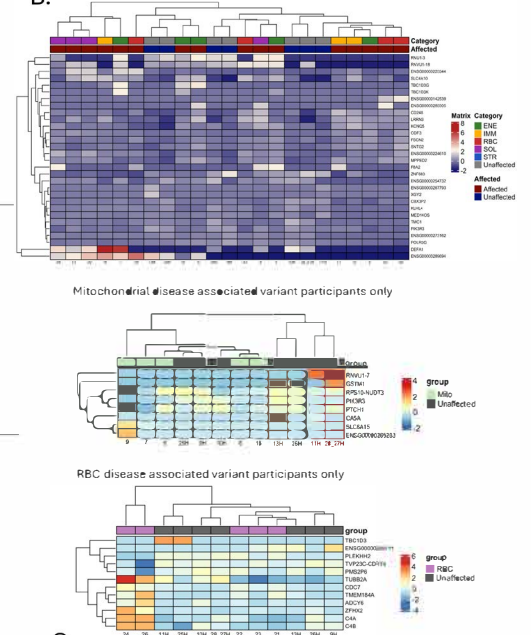

C.

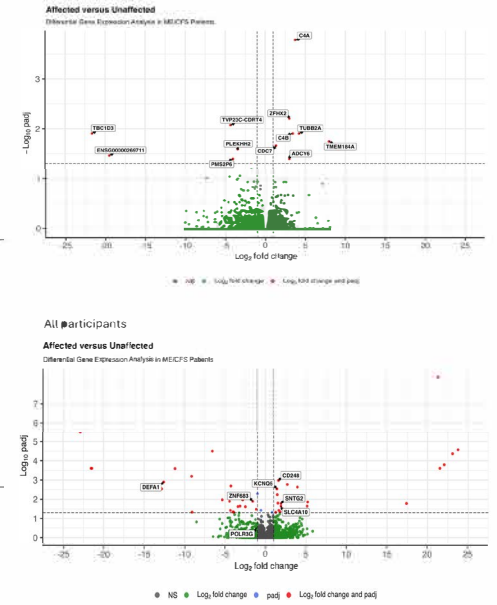

Supplement: Supplementary file 4 — Supplementary Material 4 [file 12967_2025_7586_MOESM4_ESM.pdf]

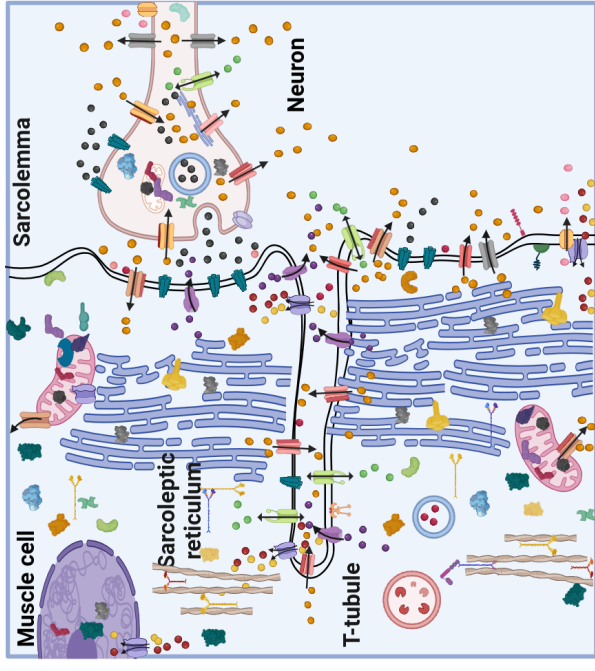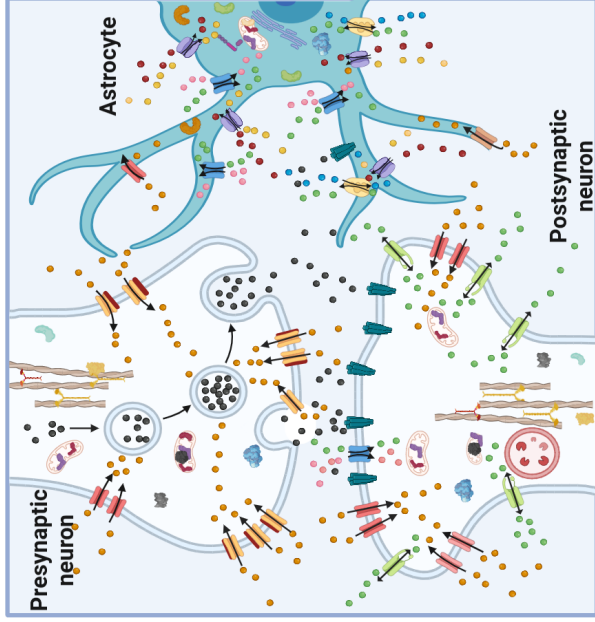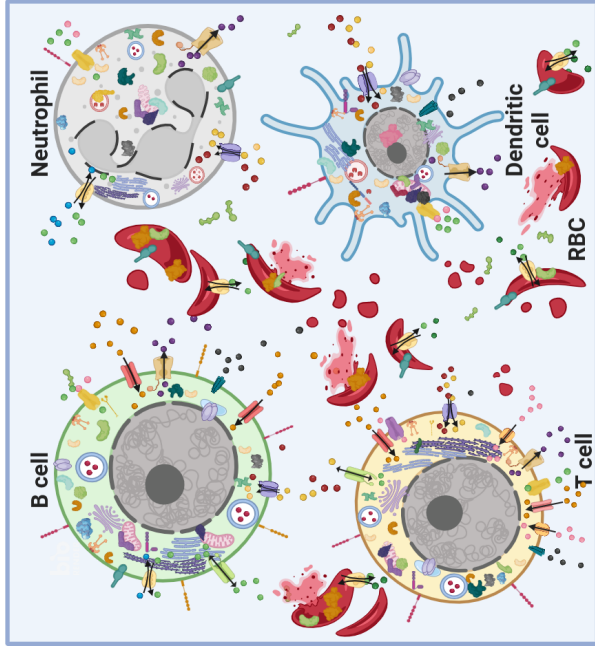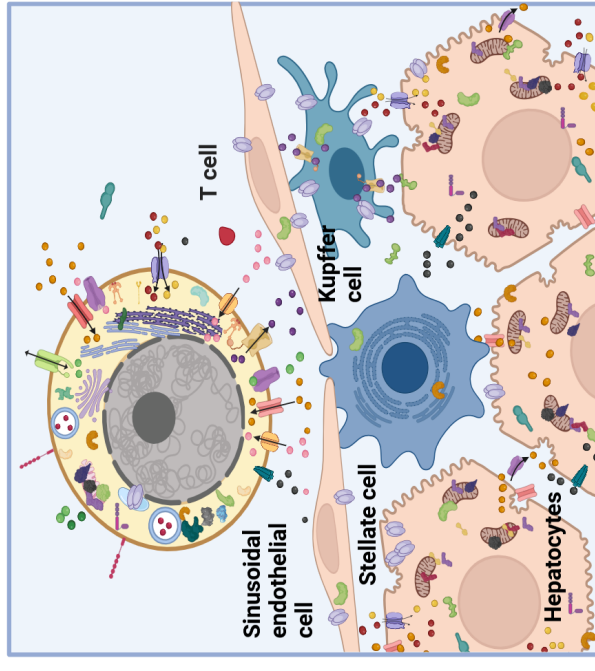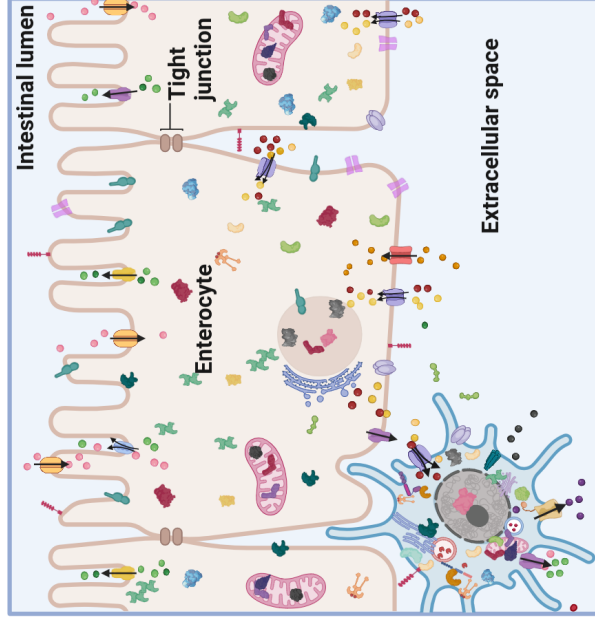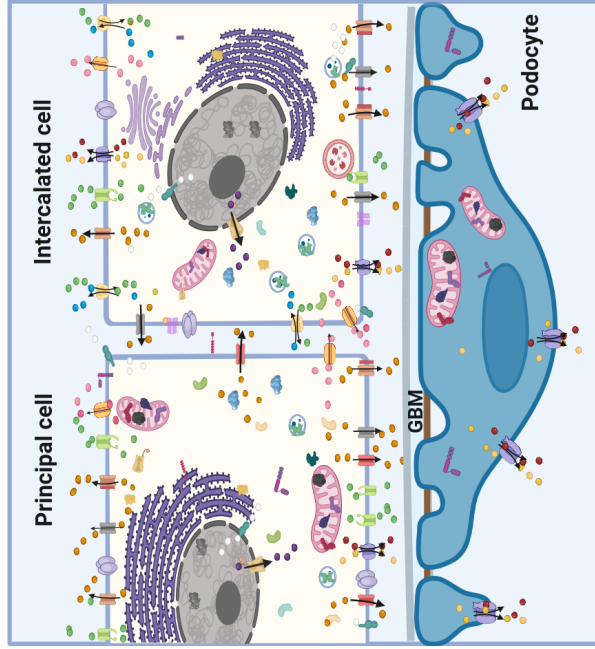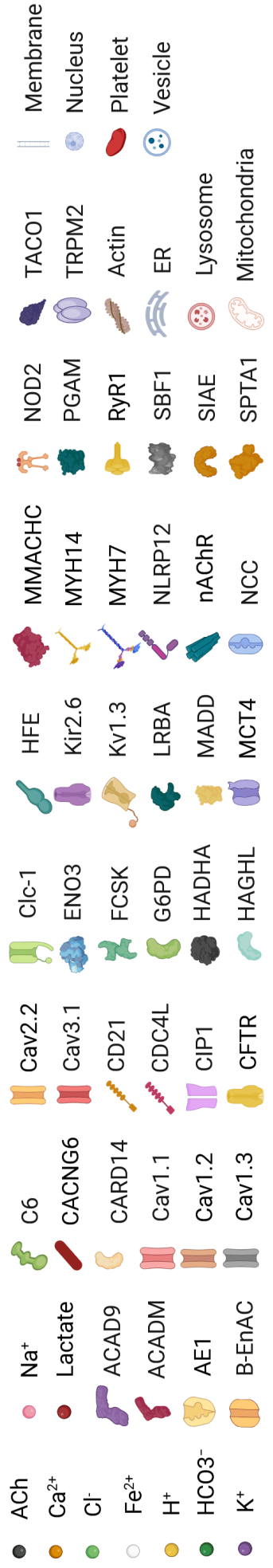

Supplement: Supplementary file 5 — Supplementary Material 5 [file 12967_2025_7586_MOESM5_ESM.pdf]
