# Supplementary material for: Uncovering the genetic architecture of ME/CFS: a precision approach reveals impact of rare monogenic variation
Source: J Transl Med. 2025 Dec 24;24:168. doi: 10.1186/s12967-025-07586-w (PMC12888368; doi:10.1186/s12967-025-07586-w)
Supplement: Supplementary file 3 — Supplementary Material 3 [file 12967_2025_7586_MOESM3_ESM.pdf]

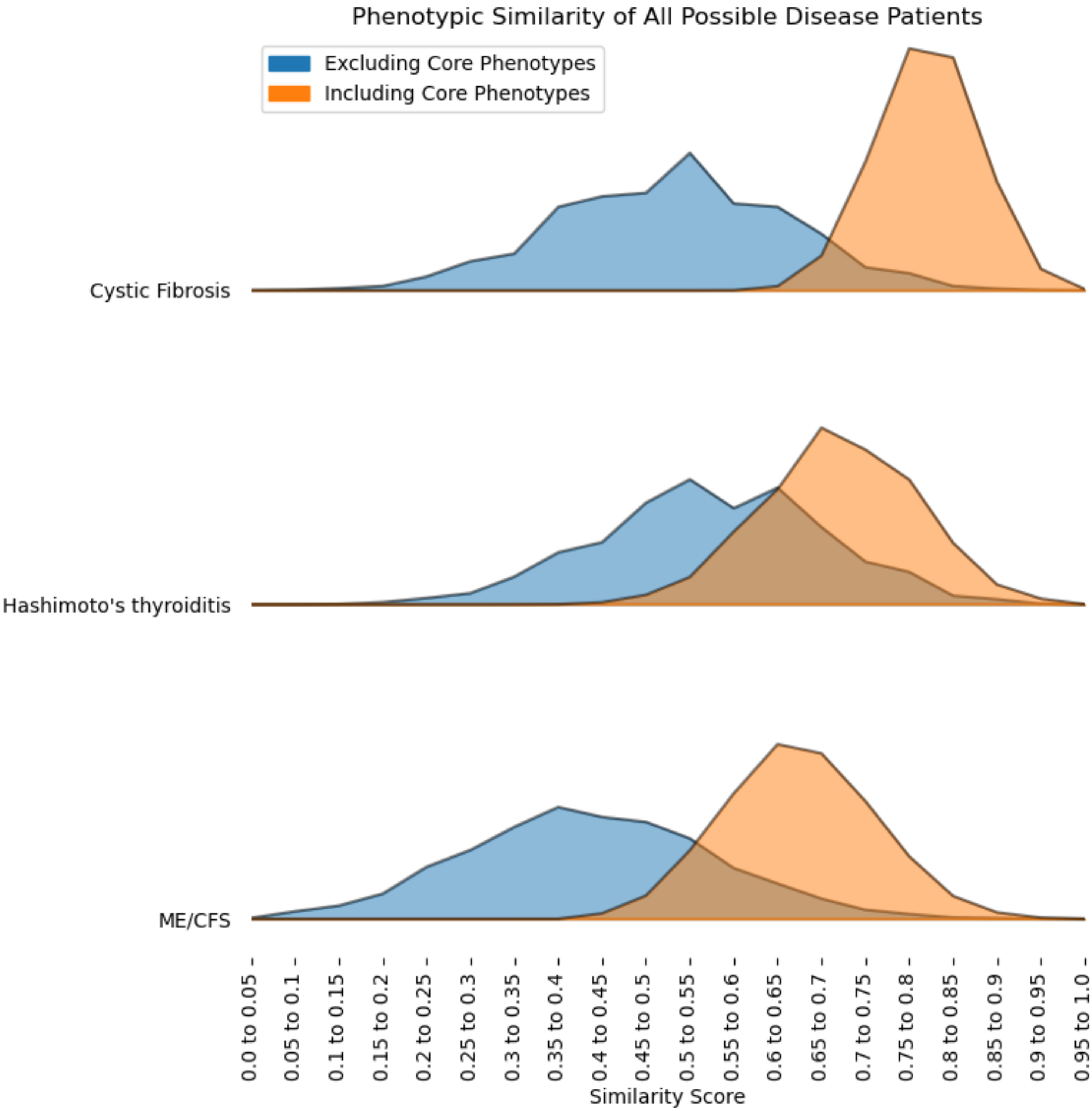

Phenotypic Similarity of All Possible Disease Patients
Excluding Core Phenotypes
Including Core Phenotypes
Cystic Fibrosis
Hashimoto's thyroiditis
ME/CFS
0.0 to 0.05
0.05 to 0.1
0.1 to 0.15
0.15 to 0.2
0.2 to 0.25
0.25 to 0.3
0.3 to 0.35
0.35 to 0.4
0.4 to 0.45
0.45 to 0.5
0.5 to 0.55
0.55 to 0.6
0.6 to 0.65
0.65 to 0.7
0.7 to 0.75
0.75 to 0.8
0.8 to 0.85
0.85 to 0.9
0.9 to 0.95
0.95 to 1.0
Similarity Score
