# Supplementary material for: Uncovering the genetic architecture of ME/CFS: a precision approach reveals impact of rare monogenic variation
Source: J Transl Med. 2025 Dec 24;24:168. doi: 10.1186/s12967-025-07586-w (PMC12888368; doi:10.1186/s12967-025-07586-w)
Supplement: Supplementary file 6 — Supplementary Material 6 [file 12967_2025_7586_MOESM6_ESM.docx]

**Supplemental Table 1. Exclusionary criteria for ME/CFS participants**

| Diagnosis of an autoimmune disorder other than thyroid disease |
| --- |
| Uncontrolled thyroid disorder |
| Type I or Type II diabetes |
| Diagnosed neurological or neurodegenerative condition |
| Blood clotting disorder |
| Positive rheumatoid factor (RF) |
| C-Reactive Protein over 10mg/dL |
| Complete blood count indicating abnormal immune function |
| Complete blood count indicating anemia |
| Complete blood count indicating cancer |
| Anti-nuclear antibody ratio over 1:80 |
| Erythrocyte sedimentation rate over 60mm/hr. |
| Impaired renal or hepatic function |
| Hospital Anxiety and Depression Scale (HADS) depression subscale score over 16 |
| Significant psychiatric condition impairing ability to follow study procedures |
| Use of anticoagulants |
| Use of any prescription medication indicating the presence of an exclusionary comorbidity |
